# The Initial Learning Curve for Robot-Assisted Sleeve Gastrectomy: A Surgeon's Experience While Introducing the Robotic Technology in a Bariatric Surgery Department

**DOI:** 10.1155/2012/347131

**Published:** 2012-09-17

**Authors:** Ramon Vilallonga, José Manuel Fort, Oscar Gonzalez, Enric Caubet, Angeles Boleko, Karl John Neff, Manel Armengol

**Affiliations:** ^1^Endocrine, Metabolic and Bariatric Unit, General Surgery Department, Vall d'Hebron University Hospital, Center of Excellence for the EAC-BC, Passeig de la Vall d'Hebron 119-129, 08035 Barcelona, Spain; ^2^General Surgery Department, Vall d'Hebron University Hospital, Passeig de la Vall d'Hebron 119-129, 08035 Barcelona, Spain; ^3^Department of Experimental Pathology, University College Dublin, Dublin, Ireland

## Abstract

*Objective*. Robot-assisted sleeve gastrectomy has the potential to treat patients with obesity and its comorbidities. To evaluate the learning curve for this procedure before undergoing Roux en-Y gastric bypass is the objective of this paper. *Materials and Methods*. Robot-assisted sleeve gastrectomy was attempted in 32 consecutive patients. A survey was performed in order to identify performance variables during completion of the learning curve. Total operative time (OT), docking time (DT), complications, and length of hospital stay were compared among patients divided into two cohorts according to the surgical experience. Scattergrams and continuous curves were plotted to develop a robotic sleeve gastrectomy learning curve. *Results*. Overall OT time decreased from 89.8 minutes in cohort 1 to 70.1 minutes in cohort 2, with less than 5% change in OT after case 19. Time from incision to docking decreased from 9.5 minutes in cohort 1 to 7.6 minutes in cohort 2. The time required to dock the robotic system also decreased. The complication rate was the same in the two cohorts. *Conclusion*. Our survey indicates that technique and outcomes for robot-assisted sleeve gastrectomy gradually improve with experience. We found that the learning curve for performing a sleeve gastrectomy using the da Vinci system is completed after about 20 cases.

## 1. Introduction

The sleeve gastrectomy (SG) is the first part of the duodenal switch operation and leaves a lesser curvature tube after excising the fundus and greater curvature portion of the stomach. This surgery has become more and more popular as the first stage in the treatment of obesity [1, 2]. Minimally invasive surgery is being incorporated into general surgical practice. During the last decade, the advent of the da Vinci Surgical System (Intuitive Surgical, Sunnyvale, CA, USA) has enabled many complex procedures to be performed with minimally invasive techniques in bariatric surgery [3]. Roux en Y gastric bypass remains one of the most challenging procedures performed by bariatric and general surgeons [4]. Sleeve gastrectomies are a less technically demanding procedure, and for this reason, we used them to gain dissection and suturing experience using the da Vinci system. This initial experience was used to determine the learning curve in performing the robot-assisted sleeve gastrectomy.

## 2. Materials and Methods

Between February 2010 and April 2011, a trained surgeon in advanced laparoscopic surgery (RV and JMF) performed 32 consecutive robotic sleeve gastrectomies (RSGs) for the treatment of morbid obesity. Patients were included according to the waiting list inclusion and all meet the criteria for sleeve gastrectomy. The surgical team consisted of two attending physicians who shared the console and the scrubbed table activities. R. Vilallonga trained in a pig model performing 10 nephrectomies prior to beginning the RSG. The two surgeons worked consistently within the same roles; R. Vilallonga was in the console and J. M. Fort at the patient's side in all cases. The study adhered to all ethical guidelines considered in our institution.

### 2.1. Pneumoperitoneum and Trocar Placement

**Figure 1:**
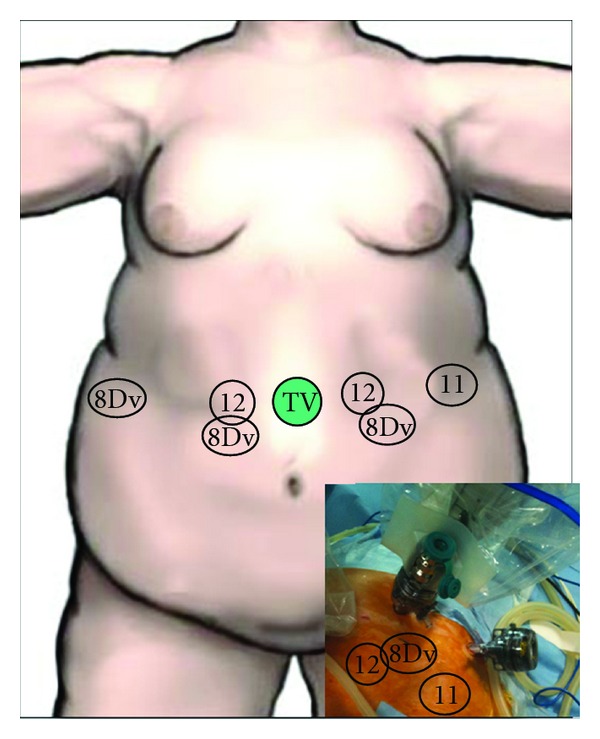


The Veress needle technique was used to establish the pneumoperitoneum into the left hypochondrium. A 12 mm port was inserted 120 mm inferior and slightly left to the sternum for camera access. For the latter port, we used an extra large 150 mm long trocar (Xcel trocar, Ethicon-Endosurgery, Cincinnati, OH, USA). The right 12 mm working port was positioned 6 cm from the midline trocar. The left 12 mm working port was located 6 cm to the left of the midline trocar. An 11 mm trocar was placed laterally to the left hypochondrium (to allow the table assistant to assist and also to place the left arm of the robot during surgery) and an 8 mm da Vinci trocar was placed under the right hip as laterally as possible (anterior axillary line) to allow liver retraction. The 8 mm da Vinci trocars were inserted through standard, disposable 12 mm trocars. This double-cannulation technique was used because standard 12 mm trocars are required during the insertion of the staples. All trocars were inserted under direct visualization with the da Vinci system camera (Figure 1).

At this stage of the procedure, we began recording the docking time (DT). The robotic camera was locked last but was used to insert all robotic cannulas and instruments. The robotic cart was positioned over the patient's head (which was covered with head protection designed for this purpose). Once the general setup was ready, the procedure began with the console surgeon using a grasper in the left hand and a modified harmonic scalpel in the right hand. The third da Vinci arm used another forceps in order to retract the liver from the 8 mm trocar placed in the right-hand side of the patient. The greater curvature of the stomach was sectioned at the lowest point in order to reach the lesser epiploic sac. This stage of the procedure is completely robotic and the first assistant does not usually participate. The division of the gastrocolic and gastrosplenic ligament continued exactly as in a standard LSG. The robot ensures precision in the upper part of the stomach, in which you need to avoid any injury to the spleen and properly visualize the vessels. Dissection continued up to 5 cm from the pylorus following dissection of the upper part of the stomach.

### 2.2. Sleeve Calibration, Section, and Extraction

At this stage of the procedure, the anaesthesiologist inserted a 32 Fr bougie to calibrate the sleeve. The anesthesiologist did not encounter any difficulty placing the bougie with the robotic bedside cart. A stapler (Echelon 60 Endopath stapler, endoscopic linear cutter straight, Ethicon-Endosurgery, Cincinnati, OH, USA), loaded with a green cartridge, was used to divide the stomach from the lowest tip of the greater gastric curvature, 5 cm proximally to the pylorus, towards the lateral edge of the bougie. This manoeuvre was performed twice. The right arm was again docked and the left robotic arm was switched to the left lateral 11 mm trocar. This manoeuvre allowed the decannulation of the right arm from the 12 mm trocar without moving the robot and can be performed within a few seconds. The table surgeon inserted a stapler loaded with blue cartridges in order to divide the sleeve up to the end of the upper part. The stomach was then removed from the cavity through the 12 mm trocar. A robotic continuous polypropylene suture (3/0) (Prolene, Ethicon-Endosurgery) was used to oversew the entire sleeve staple line. A robotic needle holder was used for this purpose. The anaesthesiologist filled the sleeve with diluted methylene blue in order to detect any leakage from the staple line.

### 2.3. Postoperative Management and Followup

The nasogastric tube was removed on postoperative day one. All patients underwent a mandatory upper gastrointestinal tract series with contrast material on the third postoperative day. If this was normal, patients were discharged. All patients received nutritional advice and were instructed to follow a liquid and semiliquid diet for 2 weeks. All patients are followed up every 3 months in the outpatient clinic until the end of the second postoperative year and then are seen annually.

### 2.4. Learning Curve and Data Management

A retrospective review of our prospective obesity surgery database was conducted. Variables examined included overall operative time, docking time, length of hospital stay, and complications. Continuous curves were plotted for each variable to identify any plateau effect. The patient number at which a <5% change occurred within a variable gave the minimum number of cases needed to reach the learning curve for that variable. In order to examine the learning curve associated with selected continuous endpoints as the number of operative cases increased, a negative exponential model was fitted via least squares estimation. This model represents the estimated plateau. 

## 3. Results

Robot-assisted sleeve gastrectomy was performed in 32 patients, of whom 12 were males and 20 females. Their mean age was 44 years, and the mean BMI was 48.3 kg/m². 8 patients had diabetes, 13 had hypertension, 9 patients had dyslipidemia, and 16 were using a continuous positive airway pressure (CPAP) device at home at the time of operation. There were no differences between the two cohorts in terms of BMI (Table 1).

All patients were included consecutively according to the waiting list order and the eligibility for sleeve gastrectomy. From the first 12 cases that configured cohort 1, there were 3 males and 9 females. Of all 32 patients, none required laparoscopic or open conversion. The set-up time gradually decreased to 34.9 minutes as the nurses became more experienced. Two laparoscopic and robotic operating tables were always prepared and preparation of the robot was included in this set-up time.

**Figure 2:**
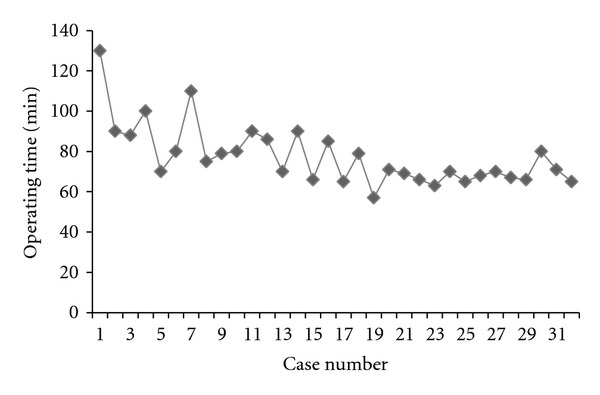


**Figure 3:**
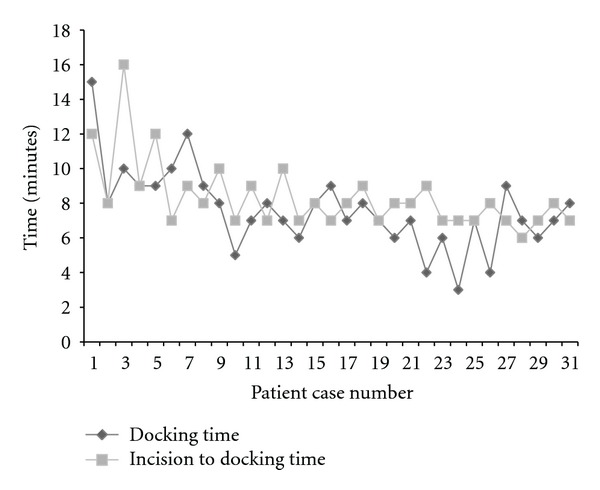


The overall operating time (OT) decreased from 89.8 minutes in cohort 1 to 70.1 minutes in cohort 2; there was less than 5% change in OT after case 19 up to case 32 (Figure 2). This decrease in OT was attributed to better understanding of the technique and the development of a coordinated procedure. The average time from incision to docking the robot was 8.8 minutes. However, time from incision to docking decreased from 9.5 minutes in cohort 1 to 7.6 minutes in cohort 2. The time taken to dock the robotic system also decreased from 9.1 minutes in cohort 1 to 6.6 minutes in cohort 2. The complication rate was comparable between the two cohorts (Table 2). The plateau on the curve for time from incision to docking, docking, and total operative time occurred at the 19th–22nd patient with <5% change from this point (Figure 3). The followup was uneventful for all patients in terms of nausea, vomiting, or stenosis, with a mean followup of 10 months.

## 4. Discussion

Sleeve gastrectomy is a purely restrictive operation that reduces the size of the gastric reservoir to 60–100 mL, permitting the intake of only small amounts of food and imparting a feeling of satiety earlier during a meal. It has been performed laparoscopically with good results [1]. In 2000, the Food and Drug Administration (FDA) approved the da Vinci Surgical System (Intuitive Surgical Inc, Sunnyvale, CA, USA) for use in general laparoscopic surgery, and since then many surgeons have used this system in order to improve their surgical outcomes [5]. It has also been used in bariatric surgery to complete demanding surgeries such as GBP, which requires high levels of expertise even in trained surgeons [6, 7].

Our data support the conclusion that both setup and docking of the robot can be achieved within an acceptable time after the learning curve. The learning curve process may have a low impact on overall surgical time. However this can only be determined by comparing subsequent cases with the first cases performed by each surgeon. Unfortunately, the relevant data were not available. Set-up time and docking time were recently evaluated for different robotic surgeries, and it was shown that they could be initially time consuming but that they are easy to learn and have steep learning curves [8]. The same was found in our initial experience working with the same scrub-nurse team and the same surgical team members. 

No data are available on the learning curve for robotic sleeve gastrectomy. Also, we have not been able to compare the learning curve of RSG procedure to RGBP because only 7 cases have been performed. Laparoscopic sleeve gastrectomy can be safely and efficiently performed in a newly established bariatric centre following a mentorship procedure. Extended mentoring has been shown to affect outcomes, especially for less experienced surgeons [9]. It is known that sleeve gastrectomy is a less technically demanding procedure compared to gastric bypass. However, when implementing new technologies such as robotic assisted surgery, it can be a more amenable procedure than gastric bypass. 

In addition, the learning curve has been reported to be shorter for surgeons who initiated their experience at an institution with an established laparoscopic bariatric programme [10, 11]. A learning curve can be identified in operative times and complications. Some authors have shown that proficiency seems to require 68 cases [12]. We included more patients in order to determine the number of cases needed to produce a plateau in these variables. 

Some previous articles have suggested that it took 30 robotic cases to perform the procedure in less time than it took for her median laparoscopic times. They, therefore, concluded that the learning curve was 30 cases [13]. Buchs in his article “*Learning curve for robot-assisted Roux-en-Y gastric bypass*” assessed the learning curve using a cumulative sum method. He found the learning curve consisted of two distinct phases: phase 1 (the initial 14 cases; mean OT, 288.9 min) and phase 2 (the subsequent cases; mean OT, 223.6 min), which represented the mastery phase, with a decrease in OT (*P* = 0.0001) [14]. However there could be a phase three, a phase four, or even beyond as in our case series. His mean operative time of 223.6 min for phase 2 (his defined mastery phase), which is considerably longer than typical operative times for robotic gastric bypass, makes it very likely that there are more stabilization points in operative time. For this reason, we have to comment that we could have a sample size too small to capture this stabilization phenomenon. However, times and result in terms of complications, outcomes, and results are satisfactory.

When discussing robotics, all authors are concerned about time. It is clear that time can be a major issue in robotic surgery. For this reason, we focused specifically on the set-up and docking times of the da Vinci surgical system in order to perform surgery efficiently. 

According to our data, trained nurses can achieve robotic setup efficiently, and docking can be conducted time effectively by the console surgeon and the first assistant. As shown previously, a trained nurse can complete robotic draping within 35 minutes while the patient is in preparation for anesthesia. The learning curve for docking has been successfully completed in our experience. Some authors have observed an increase in operating time when using the robotic system, but we believe that a learning curve is required in order to decrease time loss and potential risks [15–17]. 

To our knowledge the only previous report of robotic sleeve gastrectomy mentioned the advantages of using this procedure instead of a robotic gastric bypass (RGBP) as the first step to introducing robotic surgery to a bariatric unit [18]. They suggested, and we agree, that it is always wiser to start with a less demanding procedure in order to avoid errors in the initial phases of the overall robotic learning curve. In this paper, no data were reported concerning the learning curve before attempting to undergo a RYGBP. In our experience, and according to our protocol, we perform sleeve gastrectomy in superobese patients (BMI > 50) and we consider it more suitable for initial robotic training. Using robotic assisted techniques, even in part, could be considered in RYGBP during a learning curve instead of reinforced staple line RSG. Robotic assisted RYGBP was recently performed effectively in more than 300 patients [19]. However, we suggest that RSG be completed before RYGBP is introduced to routine clinical practice within a bariatric unit.

## 5. Conclusion

Our early experience in RSG suggests that robotic surgery is safe, feasible, and could be an effective alternative to the conventional laparoscopic approach in bariatric surgery. Robotic surgery gives all the benefits of the laparoscopic approach, with added benefits in certain challenging surgical cases. However, we believe that bariatric surgeons should be trained in RSG before RYGBP.

Completion of a learning curve is mandatory even in experienced laparoscopic surgeons before undergoing technically demanding robotic procedures such as the RYGBP. Despite the lack of tactile feedback, the long set-up time, long learning curve, and continued high costs, robotic systems can be used in particularly challenging surgeries. According to our criteria and our results, the learning curve for a console surgeon for sleeve gastrectomy should be completed by around 20 cases. Once this point has been reached and the operator is confident in suturing and docking with the robot, more challenging techniques can be considered. In our experience, sleeve gastrectomy can be achieved safely and could be considered as a preliminary step prior to attempting more complex bariatric procedures through a robotic assisted approach. However, partial RGBP may also be reasonable as an initial procedure.

## Figures and Tables

**Table 1:** Demographic data.

	All patients (*n* = 32)
Mean age (yrs.), min–max	44.7, 24–61
Sex (M : F)	12 : 20
Mean BMI (Kg/m^2^), SD	48.3, 6.2
Diabetes mellitus (*n*)	8
HTA (*n*)	13
Dyslipidemia (*n*)	9

**Table 2:** Operating times and postoperative data.

	All patients (*n* = 32)	Cohort 1 (*n* = 12)	Cohort 2 (*n* = 20)
Mean total set-up time (min), min–max	36.5, 25–60	38.0, 25–60	34.9, 30–45
Mean incision to docking time (min), min–max	8.3, 7–16	9.5, 7–16	7.6, 7–10
Mean total docking time (min), min–max	7.9, 5–15	9.1, 5–15	6.6, 3–8
Mean time for sleeve gastrectomy (min), min–max	77.5, 56–130	89.8, 70–130	70.1, 56–90
Complications (*n*), cause	1, postop. abscess drained by CT scan.	1	0
